# Tracking SARS-CoV-2 lineage B.1.1.7 dissemination: insights from nationwide spike gene target failure (SGTF) and spike gene late detection (SGTL) data, Portugal, week 49 2020 to week 3 2021

**DOI:** 10.2807/1560-7917.ES.2021.26.10.2100130

**Published:** 2021-03-11

**Authors:** Vítor Borges, Carlos Sousa, Luís Menezes, António Maia Gonçalves, Miguel Picão, José Pedro Almeida, Margarida Vieita, Rafael Santos, Ana Rita Silva, Mariana Costa, Luís Carneiro, Pedro Casaca, Pedro Pinto-Leite, André Peralta-Santos, Joana Isidro, Sílvia Duarte, Luís Vieira, Raquel Guiomar, Susana Silva, Baltazar Nunes, João P Gomes

**Affiliations:** 1 Bioinformatics Unit, Department of Infectious Diseases, National Institute of Health Dr. Ricardo Jorge (INSA), Lisbon, Portugal; 2 These authors contributed equally to this work; 3 Molecular Diagnostics Laboratory, Unilabs, Oporto, Portugal; 4 Executive Office, Unilabs, Oporto, Portugal; 5 Medical Office, Unilabs, Oporto, Portugal; 6 IT Office, Unilabs, Oporto, Portugal; 7 Data Intelligence, Unilabs, Oporto, Portugal; 8 Division of Epidemiology and Statistics, Directorate of Information and Analysis, Directorate-General of Health, Lisbon, Portugal; 9 Innovation and Technology Unit, Department of Human Genetics; National Institute of Health Dr. Ricardo Jorge (INSA), Lisbon, Portugal; 10 National Reference Laboratory for Influenza and other Respiratory Viruses, Department of Infectious Diseases; National Institute of Health Dr. Ricardo Jorge (INSA), Lisbon, Portugal; 11 Epidemiological Research Unit Department of Epidemiology, National Institute of Health Dr. Ricardo Jorge (INSA), Lisbon, Portugal

**Keywords:** SARS-CoV-2, B.1.1.7 lineage, RT-PCR, spike gene target failure (SGTF), spike gene target late detection (SGTL), laboratory surveillance, genome sequencing

## Abstract

We show that the SARS-CoV-2 B.1.1.7 lineage is highly disseminated in Portugal, with the odds of B.1.1.7 proportion increasing at an estimated 89% (95% confidence interval: 83–95%) per week until week 3 2021. RT-PCR spike gene target late detection (SGTL) can constitute a useful surrogate to track B.1.1.7 spread, besides the spike gene target failure (SGTF) proxy. SGTL/SGTF samples were associated with statistically significant higher viral loads, but not with substantial shift in age distribution compared to non-SGTF/SGTL cases.

The severe acute respiratory syndrome coronavirus 2 (SARS-CoV-2) lineage B.1.1.7, also designated variant of concern (VOC) 202012/01 or 501Y.V1, has shown a pronounced frequency increase in the United Kingdom (UK) since November 2020 and has rapidly expanded its geographical range worldwide [1-5]. The SARS-CoV-2 B.1.1.7 lineage harbours a 21765–21770 genomic deletion (spike Δ69–70) that affects the detection of the spike (S) gene by some real-time polymerase chain reaction (RT-PCR) assays (e.g. TaqPath COVID-19, ThermoFisher, Waltham, MA, United States, targeting N, ORF1ab and S genes), leading to what has been termed ‘spike gene target failure (SGTF)’ or ‘spike gene drop out’ [1,6]. This coincidental occurrence has provided a good proxy for monitoring trends of B.1.1.7 [1,7-10].

We investigated the proportion of SGTF cases to gain insight on B.1.1.7 frequency and geographical spread in Portugal.

A detailed description of the Material and Methods is presented in the Supplement, including RT-PCR procedures and rationale for sample classification, genome sequencing, bioinformatics and statistical analysis.

## Spike gene target failure and spike gene target late detection RT-PCR data as proxies for monitoring B.1.1.7 circulation

We took advantage of a large SARS-CoV-2 TaqPath COVID-19 RT-PCR data set comprehensively collected at the community level between week 49 2020 and week 3 2021 by Unilabs (Oporto), a large private laboratory with 287 testing sites distributed throughout the country.

We assumed that SGTF can be a reliable indicator of B.1.1.7 circulation in our country, considering that: (i) Portugal was among the top destinations for air travellers from the UK during early autumn [11]; (ii) the laboratories at the Portuguese National Institute of Health Dr. Ricardo Jorge (INSA) detected multiple independent B.1.1.7 introductions since early December 2020, among the B.1.1.7-associated cases confirmed by sequencing [12], as at 5 February 2021 and (iii) B.1.1.7 was identified by sequencing in 91.9% (79/86) of known SGTF-positive samples up to week 3 2021.

Between week 49 2020 and week 3 2021, Unilabs performed 170,658 SARS-CoV-2 RT-PCR tests using the TaqPath COVID-19 assay, which roughly corresponds to 8% of all SARS-CoV-2 RT-PCR tests done in Portugal in the same period. Of the 36,651 positive results, 3,367 (9.2%) corresponded to SGTF tests, as defined by a positive test with non-detectable S gene and cycle threshold (Ct) values of ≤ 30 for N and ORF1ab targets. During the same period, we also unexpectedly detected that in 1.5% (561/36,651) of TaqPath COVID-19 positive samples Ct values for the S gene were > 5 units higher than the maximum Ct value obtained for the other two targets (N and ORF1ab) of the assay (again, counts only included positive samples with ≤ 30 Ct for N and ORF1ab targets). The mean Ct difference between S gene and N/ORF genes for these samples was consistently around 5–8 Ct values (median: 6.60; interquartile range (IQR): 5.79–7.59), so we tentatively designated this RT-PCR profile as ‘spike gene target late detection’ (SGTL).

The delayed detection (i.e. higher Ct values) might be due to stochastic and low frequent probe misannealing, rendering detectable PCR signals only when a high number of S target amplicons are present in the reaction. B.1.1.7 was identified by sequencing in 11 of 12 SGTL positive samples, with the 21765–21770 genomic deletion (spike Δ69–70) being finely inspected and unequivocally confirmed in all SGTL samples. These data, together with the parallel scenario observed for SGTF and STGL regarding frequency increase and viral loads (see below), supports that SGTL can constitute an additional proxy to detect and monitor B.1.1.7 lineage. This phenomenon of late S gene detection has been observed in England, and diagnostic laboratories have been advised of such possible alternative presentation of B.1.1.7 [13].

## Continued increase in the proportion of spike gene target failure and spike gene target late detection samples

**Figure 1:**
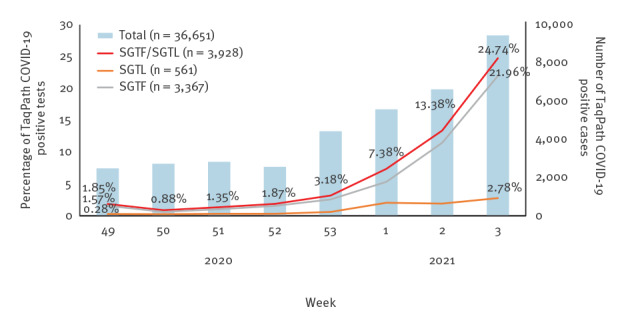
Proportion of spike gene target failure and spike gene late detection positive samples among all TaqPath COVID-19 positive samples, Portugal, week 49 2020–week 3 2021

COVID-19: coronavirus disease; Ct: cycle threshold; SARS-CoV-2: severe acute respiratory syndrome coronavirus 2; SGTF: spike gene target failure; SGTL: spike gene late detection.

**Figure 2:**
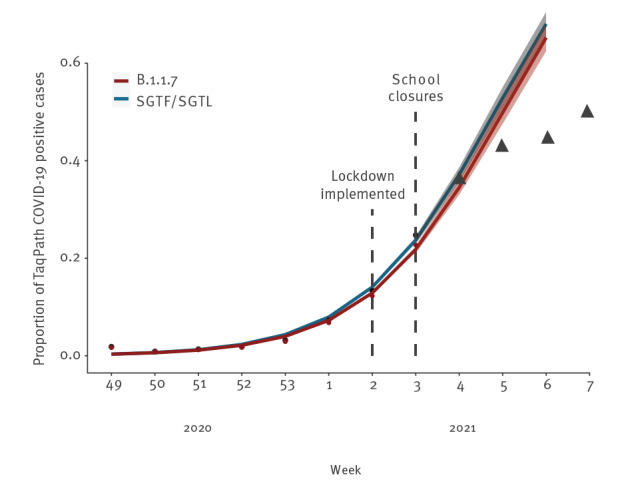
Estimated weekly frequency time trend of spike gene target failure/spike gene target late detection and B.1.1.7 cases using a binomial logistic model with 95% prediction interval, Portugal, week 3–week 6 2021

COVID-19: coronavirus disease; SGTL: spike gene late detection; SGTF: spike gene target failure.

The proportion of both SGTF and SGTL cases has continually risen since the beginning of December (week 49 2020) (Figure 1), reaching a total of 22.0% and 2.8% of all TaqPath COVID-19 positive samples, respectively, in week 3 2021. Since week 53 2020, the aggregated proportion of SGTF plus SGTL (hereafter defined as SGTF/SGTL) cases increased eightfold, reaching 24.7% in week 3 2021. The odds of SGTF/SGTL proportion increased at a 90% (95% confidence interval (CI): 85–96) rate per week. We forecast—assuming no change in the increasing rate—that the proportion of SGTF/SGTL cases can reach up to 68% (95% CI: 65–71) of positive cases by week 6 (Figure 2).

SGTF refers to  positive test with non-detectable S gene Ct value  and Ct ≤ 30 for N and ORF1ab targets.

SGTL  refers to  positive test having Ct values for S gene > 5 units higher than the maximum Ct value obtained for the other two targets (N and ORF1ab) of the assay (exclusively includes positive samples with ≤ 30 Ct for N and ORF1ab targets).

The data set was comprehensively collected at the community level by Unilabs, a large laboratory distributed throughout Portugal. It comprises ca 8% of all SARS-CoV-2 RT-PCR tests done in Portugal in the same period.

SGTF refers to  positive test with non-detectable S gene Ct value  and Ct ≤ 30 for N and ORF1ab targets.

SGTL  refers to  positive test having Ct values for S gene > 5 units higher than the maximum Ct value obtained for the other two targets (N and ORF1ab) of the assay (exclusively includes positive samples with ≤ 30 Ct for N and ORF1ab targets).

Black and red dots indicate the observed and estimated proportion of SGTF/SGTL and B.1.1.7 cases used in the projection (data collected between week 49 2020 and week 3 2021), respectively, while triangles denote the observed proportion of SGTF/SGTL cases beyond the study period (week 4–week 7 2021). The shaded areas indicated the 95% confidence interval for the SGTL/SGTF and B.1.1.7 estimates.

The proportion of SGTF/SGTL cases that are B.1.1.7 was estimated as 0.918 (95% CI: 0.845–0.964) based on a sub-sample of sequencing cases (90 B.1.1.7 cases in 98 known SGTF/SGTL sequenced samples). Thus, assuming this constant proportion of 0.918, the corresponding estimates for B.1.1.7 lineage are: (i) a proportion among SARS-CoV-2 detections of 12.3% and 22.7% at weeks 2 and 3, (ii) a 89% (95% CI: 83–96) odds increase rate per week and (iii) a forecast of a proportion of 65% (95% CI: 62–68) among SARS-CoV-2 detections on week 6 (Figure 2). This high growth rate (almost doubling each week) parallels what has been reported in other countries, namely in the UK [3] and Denmark [14]. Of note, given that the frequency of SGTF/SGTL that are B.1.1.7 is expected to increase over time (as observed in the UK [13]), we do not rule out that there is a potential bias in our estimates because of the use of a constant value (0.918) for the B.1.1.7 proxy.

Nevertheless, supporting the accuracy of the application of SGTF/SGTL as a proxy for B.1.1.7 and of the estimated B.1.1.7 relative frequency in Portugal during the study period, recent sequencing of 495 randomly collected SARS-CoV-2 RT-PCR positive samples across the country during week 2 identified 14.5% of B.1.1.7 sequences [12].

As shown in Supplementary Figure S1, SGTF/SGTL cases are dispersed throughout mainland Portugal, indicating that the B.1.1.7 lineage is highly disseminated and there is active community transmission. A general lockdown was implemented in week 2 2021, raising expectations about the impact that the expected reduction in the number of COVID-19 cases would have on the increasing rate of the relative frequency of the B.1.1.7 lineage. After the increase in frequency observed during the last week of 2020 and the first 3 weeks of 2021, and upon the implementation of public health measures, we observed a decelerating trend, with the SGTF/SGTL proportion remaining below 50% in week 7 2021.

## Spike gene target failure and spike gene target late detection samples are associated with higher viral loads

**Figure 3:**
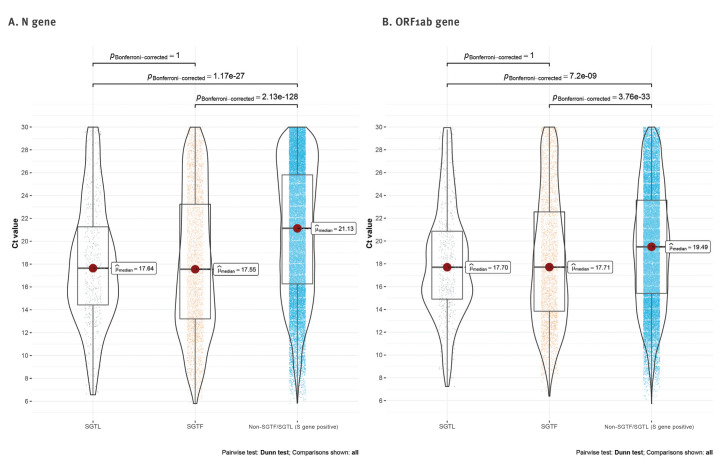
Scatter, violin and box plots of the (A) N-gene and (B) ORF1ab cycle threshold values obtained for spike gene target failure and spike gene late detection samples compared with non-SGTF/SGTL positive samples, Portugal, week 49 2020–week 3 2021 (n = 30,407)

We also investigated RT-PCR Ct values in SGTF and SGTL vs non-SGTF/SGTL positive samples. Both SGTF and SGTL samples had significantly lower median Ct values of N and ORF1ab gene targets (ca 3.5 and 1.8 Ct units, respectively) compared with samples where S gene was unbiasedly detected (Figure 3). This observation not only corroborates previous findings that B.1.1.7 SGTF samples are more likely to present higher viral loads [2,7], but also supports our finding that SGTL might be an additional surrogate to identify Δ69–70-bearing variants.

Ct: cycle threshold.

As the SGTF/SGTL group exclusively includes positive samples with ≤ 30 Ct for N and ORF1ab targets (see definition in the Supplement), only non-SGTF/SGTL positive samples (i.e., a positive test that was neither SGTF nor SGTL) having both N and ORF1a positive signals and Ct values ≤ 30 were used in this comparison. Median Ct is shown by a black horizontal line and the results of tests for significant differences are shown above both plots with conventional representation. The Kruskal-Walls one-way ANOVA non-parametric test was used to assess the existence of statistically significant differences in Ct values between groups. Differences in Ct values for each pair of groups were assessed using the Dunn test adjusted for multiple comparison tests with Bonferroni correction.

## Spike gene target failure/spike gene target late detection cases are not associated with a substantial shift in age composition

**Figure 4:**
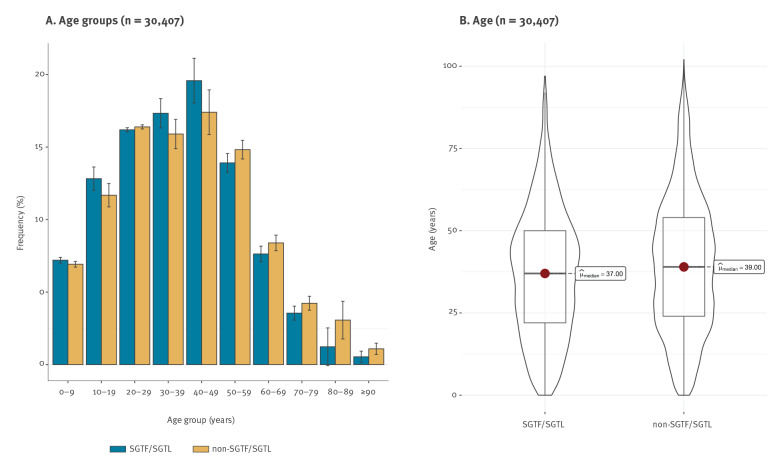
Distribution of spike gene target failure/spike gene target late detection positive cases compared with non-spike gene target failure/spike gene target late detection positive cases by (A) age groups (histogram) and (B) age (Violin and box plot), Portugal, week 49 2020–week 3 2021 (n = 30,407)

SGTF: spike gene target failure; SGTL: spike gene target late detection; Ct: cycle threshold.

Our analysis of SGTF and SGTL in different age groups did not indicate a substantial shift in the age composition when comparing them with non-SGTF/SGTL cases. In fact, although statistically different (p < 0.001), the age distributions for cases in the SGTF/SGTL (median: 37; IQR: 22–50) and non-SGTF/SGTL (median: 39; IQR: 24–53) groups did not differ substantially (Figure 4). Also, for cases in both groups, the highest frequencies were observed for individuals aged 20 to 49 years.

As the SGTF/SGTL group exclusively includes positive samples with ≤ 30 Ct for N and ORF1ab targets (see definition in the Supplement, only non-SGTF/SGTL positive samples (i.e., positive test that was neither SGTF nor SGTL) having both N and ORF1a positive signals and Ct values ≤ 30 were used in this comparison.

## Development of an interactive dashboard to aid public health decision-making

In order to facilitate real-time monitoring of SGTF/SGTL cases, an interactive dashboard and data cube, relying on Unilabs Intelli4Covid (BigData platform), was developed by Unilabs. The dashboard was shared with INSA to support timely public health decision-making by enabling early identification of geographical regions with an estimated increased incidence and circulation of lineage B.1.1.7. In fact, the release of data regarding the estimated proportion of B.1.1.7 cases up to week 2 2020 (and forecasted for the following 3 weeks) had an immediate impact, as it triggered the political decision to strengthen the ongoing confinement measures, namely through school closures in week 3 2021.

## Conclusions

In the present study, we evaluated the SARS-CoV-2 B.1.1.7 lineage dissemination in Portugal between week 49 2020 and week 3 2021. In particular, we observed that SGTL can be a proxy to identify B.1.1.7 lineage, in addition to SGTF. By almost doubling each week, the estimated B.1.1.7 proportion reached ca 23% at week 3, and at this time it was forecasted to reach 65% by week 6. Physical distancing measures implemented in weeks 2 and 3 strongly decelerated the concerning growth rate, with the proportion of SGTF and SGTL remaining below 50% until week 7 2021. In our dataset, patients whose samples exhibited either SGTF or SGTL effect in the TaqPath COVID-19 test were more likely to have high viral loads at the time of sampling. Age distribution of SGTF/SGTL cases did not seem to indicate a substantial shift in the age composition, as compared to non-SGTF/SGTL cases.

Portugal faced a high intensity transmission of SARS-CoV-2, being among the countries with the highest 14-day notification rate of newly diagnosed COVID-19 cases per 100,000 inhabitants, as of week 4 2021. Our data suggests that the highly transmissible B.1.1.7 lineage is spreading widely and progressively increasing in frequency in Portugal. This reinforces the need to implement robust public health measures adapted to this new variant in order to mitigate the impact of COVID-19 in terms of hospitalisations and deaths.

## Supplementary Data

234000 Supplement
