# Supplementary material for: Tracking SARS-CoV-2 lineage B.1.1.7 dissemination: insights from nationwide spike gene target failure (SGTF) and spike gene late detection (SGTL) data, Portugal, week 49 2020 to week 3 2021
Source: Euro Surveill. 2021 Mar 11;26(10):2100131. doi: 10.2807/1560-7917.ES.2021.26.10.2100130 (PMC7953529; doi:10.2807/1560-7917.ES.2021.26.10.2100130)

Supplement - Tracking SARS-CoV-2 lineage B.1.1.7 dissemination: insights from nationwide spike gene target failure (SGTF) and spike gene late detection (SGTL) data, Portugal, week 49 2020 to week 3 2021

Borges et al, Eurosurveillance

This supplementary material is hosted by Eurosurveillance as supporting information alongside the article "Tracking SARS-CoV-2 lineage B.1.1.7 dissemination: insights from nationwide spike gene target failure (SGTF) and spike gene late detection (SGTL) data, Portugal, week 49 2020 to week 3 2021", on behalf of the authors who remain responsible for the accuracy and appropriateness of the content. The same standards for ethics, copyright, attributions and permissions as for the article apply. Supplements are not edited by Eurosurveillance and the journal is not responsible for the maintenance of any links or email addresses provided therein.

Methods

RT-PCR SARS-CoV-2 molecular testing using TaqPath assay

This study relies on data from 170,658 SARS-CoV-2 TaqPath™ COVID 19 CE IVD RT PCR Kit (Thermo Scientific™) tests performed between week 49 (2020) and week 3 (2021) by a large laboratory (Unilabs) in 287 testing sites distributed throughout the Portuguese territory. Nasal swab samples were collected using UTM® Viral Transport (COPAN Diagnostics Inc.) at community level at these locations and were sent to the Unilabs central laboratory, in Oporto, being kept at 4ºC until processing. The time elapsed between sampling and results was always less than 24 hours. RNA extraction protocol was performed with KingFisher™ Flex instrument

and MagMAX™ Viral/Pathogen II Nucleic Acid Isolation Kit (Thermo Scientific™) according to manufacturer instructions for a 200 µL sample input. The amplification was performed on the QuantStudio™ 5 Real-Time PCR System (Applied Biosystems™). Results interpretation and data transfer to LIS (includes Cycle thresholds, Ct) performed in the COVID-19 Interpretative software (Thermo Scientific™) under HL7 communication protocol.

Cycle threshold (Ct) data obtained for the three SARS-CoV-2 gene targets (ORF1ab, N and S) was compiled for all positive tests (n = 36,651), which were classified as follows: i) "S gene target failure (SGTF), i.e., positive test with non-detectable Spike (S) gene and <=30 Ct for N and ORF1ab targets; ii) "S gene target late detection (SGTL)", i.e., positive test having Ct values for S gene >5 units higher than the maximum Ct value obtained for the other two targets (N and ORF1ab) of the assay (as for SGTF group, SGTL exclusively includes positive samples with <=30 Ct for N and ORF1ab targets); or iii) "non-SGTF/SGTL", i.e., positive test that was neither SGTF nor SGTL, regardless of the Ct range.

SARS-CoV-2 genome sequencing of SGTF and SGTL positive samples

In order to assess the proportion of SGTF/SGTL positive samples that are B.1.1.7, 86 SGTF and 12 SGTL country spread samples were subjected to SARS-CoV-2 genome sequencing at Portuguese National Institute of Health (INSA) following an amplicon-based whole-genome amplification strategy using tiled, multiplexed primers[1], according to the ARTIC network protocol (https://artic.network/ncov-2019; https://www.protocols.io/view/ncov-2019-sequencing-protocol-bbmuik6w) with slight modifications, as previously described[2]. All bioinformatics analysis (from reads quality control to variant detection/inspection, sequence consensus generation and minor variants analysis) was conducted using the online (and locally installable) INSaFLU platform (https://insaflu.insa.pt/)[3], as previously described[2]. The genome sequence of SARS-CoV-2 Wuhan-Hu-1/2019 virus (GenBank accession MN908947) was used

as reference for mapping and SNV annotation[4]. Rapid clade and lineage assignments were performed using Nextclade (https://clades.nextstrain.org/) and Phylogenetic Assignment of Named Global Outbreak Lineages (Pango lineages) (https://pangolin.cog-uk.io/)[5]. Integration of the B.1.1.7 sequences on behalf of the SARS-CoV-2 genetic diversity and geotemporal spread in Portugal can be consulted here: https://insaflu.insa.pt/covid19.

Forecast of the estimated weekly proportion of SGTF/SGTL and B.1.1.7 cases

We modelled the weekly trend of the SGTF/SGTL and B.1.1.7 frequency with a binomial logistic model (using the data collected from week 49, 2020, to week 3, 2021), given that the increase in SGTF/SGTF and B.1.1.7 frequency likely tends to plateau (i.e., the increasing rate tends to reduce with time). The estimated frequency of B.1.1.7 was inferred assuming that the proportion of SGTF/SGTL cases that are B.1.1.7 is 0.918 (0.845 to 0.964, CI 95%), based on sequencing data (90 B.1.1.7 cases in 98 known SGTF/SGTL sequenced samples analysed). Results are expressed as odds ratio (estimated increase in the odds of the outcome per unit of time), calculated as follows: frequency of "SGTF/SGTL" divided by (1 - frequency non-"SGTF/SGTL").

Comparison of ORF1a and N target Ct values (a proxy for viral load) between SGTF/SGTL and non-SGTF/SGTL positive samples.

In order to assess the viral load of SGTF/SGTL positive samples in comparison to non-SGTF/SGTL positive samples, scatter, violin and box plots were build using Ct values obtained for N and ORF1a targets for both groups. For this analysis, only samples having both N and ORF1a positive signals and Ct values <=30 were considered (30407 positive tests). The Kruskal-Walls one-way ANOVA non-parametric test was used to assess the existence of

statistically significant differences in Ct values between groups. Differences in Ct values for each pair of groups were assessed using the Dunn test adjusted for multiple comparison tests with Bonferroni correction.

Comparison of age distribution between SGTF/SGTL and non-SGTF/SGTL-associated COVID-19 positive individuals

The age (years) distribution of was plotted by age group for both SGTF/SGTL and non-SGTF/SGTL-associated individuals. As the SGTF/SGTL group exclusively includes positive samples with <=30 Ct for N and ORF1ab targets (see definition above), only non-SGTF/SGTL positive samples having both N and ORF1a positive signals and Ct values <=30 were used in this comparison. In addition, violin and box-plot were built for each group and statistical difference was assessed using the Mann–Whitney–Wilcoxon test.

References from the Methods section

1. Quick J, Grubaugh ND, Pullan ST, et al. Multiplex PCR method for MinION and Illumina sequencing of Zika and other virus genomes directly from clinical samples. *Nat Protoc* 2017; **12**(6):1261–1276. doi:10.1038/nprot.2017.066
2. Borges V, Isidro J, Cortes-Martins H, et al. Massive dissemination of a SARS-CoV-2 Spike Y839 variant in Portugal. *Emerg Microbes Infect 2020*; 2:1-58. doi: 10.1080/22221751.2020.1844552.
3. Borges V, Pinheiro M, Pechirra P, Guiomar R, Gomes JP. INSaFLU: an automated open web-based bioinformatics suite "from-reads" for influenza whole-genome-sequencing-based surveillance. *Genome Med* 2018; **10**(1):46. Published 2018 Jun 29. doi:10.1186/s13073-018-0555-0

4. Wu F, Zhao S, Yu B, et al. A new coronavirus associated with human respiratory disease in China. Nature 2020; 580(7803):E7. doi:10.1038/s41586-020-2202-3
5. Rambaut A, Holmes EC, O'Toole Á, et al. A dynamic nomenclature proposal for SARS-CoV-2 lineages to assist genomic epidemiology. Nat Microbiol 2020; 5(11):1403-1407. https://doi.org/10.1038/s41564-020-0770-5

Supplementary Figure S1. Geographical distribution of Unilabs sample collection points where SGTF and SGTL were detected between week 49 (2020) and week 3 (2021).

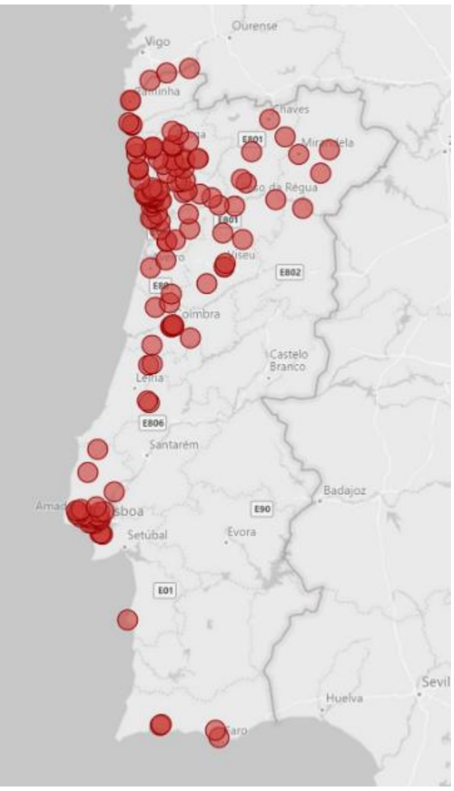

Supplement: Supplement [file 21-00131_Supplement.pdf]
